# Stress hormone-mediated antipredator morphology improves escape performance in amphibian tadpoles

**DOI:** 10.1038/s41598-021-84052-9

**Published:** 2021-02-24

**Authors:** Michael E. Fraker, Stuart A. Ludsin, Barney Luttbeg, Robert J. Denver

**Affiliations:** 1 Department of Evolution, Ecology, and Organismal Biology, The Ohio State University, Columbus, OH 43212 USA; 2 Department of Integrative Biology, Oklahoma State University, Stillwater, OK 74078 USA; 3 Department of Molecular, Cellular, and Developmental Biology and Department of Ecology and Evolutionary Biology, The University of Michigan, Ann Arbor, MI 48109 USA; 4 Present Address: Cooperative Institute for Great Lakes Research, School for the Environment and Sustainability, The University of Michigan, Ann Arbor, MI 48109 USA

**Keywords:** Community ecology, Animal physiology, Herpetology

## Abstract

Complete functional descriptions of the induction sequences of phenotypically plastic traits (perception to physiological regulation to response to outcome) should help us to clarify how plastic responses develop and operate. Ranid tadpoles express several plastic antipredator traits mediated by the stress hormone corticosterone, but how they influence outcomes remains uncertain. We investigated how predator-induced changes in the tail morphology of wood frog (*Rana sylvatica*) tadpoles influenced their escape performance over a sequence of time points when attacked by larval dragonflies (*Anax junius*). Tadpoles were raised with no predator exposure, chemical cues of dragonflies added once per day, or constant exposure to caged dragonflies crossed with no exogenous hormone added (vehicle control only), exogenous corticosterone, or metyrapone (a corticosteroid synthesis inhibitor). During predation trials, we detected no differences after four days, but after eight days, tadpoles exposed to larval dragonflies and exogenous corticosterone had developed deeper tail muscles and exhibited improved escape performance compared to controls. Treatment with metyrapone blocked the development of a deeper tail muscle and resulted in no difference in escape success. Our findings further link the predator-induced physiological stress response of ranid tadpoles to the development of an antipredator tail morphology that confers performance benefits.

## Introduction

Individuals of many taxa express predator-induced phenotypic plasticity^1,2^, yet our understanding of the underlying physiological mechanisms and subsequent performance impacts remains incomplete^3,4^. Particular aspects of the induction sequence and predator–prey interaction are well-described. For example, the production of cues of predation risk (e.g., chemical, visual, auditory), as well as how prey use them to assess risk, has received much attention^5,6^. Likewise, a growing literature has begun to identify the genetic and physiological pathways regulating phenotypic responses to predation risk^7–10^. For example, the glucocorticoid (stress hormone) signaling pathway that is similar across vertebrates has been identified as a key regulator of the antipredator phenotype across a variety of taxa^11^. Furthermore, how predator-induced trait variation affects the outcome of species interactions has been increasingly explored^12,13^. However, few studies have followed the complete induction sequence in one system from perception of the environment to physiological regulation to phenotypic response to individual outcome.

Ranid tadpoles are a model system for the study of predator-induced phenotypic plasticity for several reasons. First, chemical cues sourced from both the predator^14,15^ and prey^16^ have been identified as key information sources on risk. Second, corticosterone (CORT), a glucocorticoid stress hormone, has been shown to regulate both behavioral and morphological antipredator responses^7,16,17^. Third, induced antipredator responses are common in tadpoles^18^.

In particular, plastic tail development and morphology have been widely observed in tadpoles exposed to invertebrate predators^18,19^, but see^20^ and shown to be regulated by CORT^7^. This induced tail morphology has several hypothesized functions, which are not mutually exclusive. Increased tail muscle depth has been hypothesized to improve escape swimming velocity (i.e., startle response) during a predator attack, a hypothesis that has received mixed support^21–25^. A concurrent increase in tail surface area also may lure strikes away from the more vital parts of the body^26^, a hypothesis that has received empirical support in ranids and other genera^23,27^.

Here, we sought to clarify how the induction sequence for the plastic tail morphological response in wood frog tadpoles varies with predation risk from larval *Anax junius* dragonflies, focusing particularly on how the relationship between tail muscle depth and escape performance developed. We used a factorial design that exposed tadpoles to either (1) no predator exposure, (2) intermittent (once daily) pulses of chemical cues from larval dragonflies feeding on tadpoles, or (3) constant exposure to caged dragonflies feeding on tadpoles crossed with either (1) vehicle control, (2) exogenous CORT, or (3) the corticosteroid synthesis inhibitor metyrapone (MTP). After 0, 4, and 8 days of development, we used predation trials to test tadpole escape performance and survival time during exposure to non-caged dragonflies. We also measured behavior to begin to identify how these two broad types of plastic antipredator defenses are integrated. Based on previous results, we hypothesized that (1) constant predator exposure would result in a more rapid increase in tail muscle size than intermittent exposure, (2) CORT treatments would increase tail muscle size, while MTP would block the response, and (3) escape performance would improve concurrently in treatments in which tail muscle size increased.

## Methods

All applicable institutional and national guidelines for the care and use of animals were followed. All animal collection and experiments were approved by the Ohio State University IACUC (protocol #2016A00000028) and Ohio Division of Wildlife (Scientific Collection Permit 17-251).

### Study organisms

We collected eleven wood frog (*Rana sylvatica*) egg masses from each of four ponds (2–3 per pond) on the University of Michigan's E. S. George Reserve (Pinckney, MI, USA) during April 2018. We transported the egg masses the same day to The Ohio State University's Aquatic Ecology Laboratory (Columbus, OH, USA) and reared them in mixed groups in screen-covered outdoor pools. The pools were filled with aged, dechlorinated city water and inoculated with plankton from a local pond. We fed the tadpoles rabbit chow (16% protein) ad libitum. We collected middle-instar larval *Anax junius* dragonflies from nearby ponds, reared them individually in 475 ml containers filled with pond water, and fed each of them one tadpole three times per week prior to being used in experiments.

The day before the experiment began, we sorted tadpoles (Gosner stage 26, ~ 100–150 mg wet weight) from multiple culturing pools into sets of 60 individuals and held them in 42 × 88 × 15 cm plastic aquaria filled with 9 L of aged and dechlorinated city water. The starting density was 1 tadpole per 150 mL of water, which equaled approximately 1000 mg tadpole body weight per L of water. On Days 0, 4 and 8 we removed 9 individuals from each aquarium for body size measurements and whole body CORT analyses (described below), keeping the density the same in all treatments. We arranged all aquaria on shelves indoors under a 16:8 light:dark cycle at a constant temperature of 22 °C. That day and each day of the experiment, we added ground rabbit chow approximately equaling 10% of the total tadpole body weight to all aquaria. Each set of tadpoles was allowed to acclimate overnight.

### Induction of tail morphology

On the first day of the experiment, we hung a cage made of nylon screen on the midpoint of a side wall of each aquarium and submerged it halfway into the water column. We then randomly assigned aquaria to one of nine treatment combinations in a factorial design with four replicate aquaria for each treatment with 60 tadpoles each initially. The three predator treatments were (1) No Predator: we added 100 ml of aged, dechlorinated city water daily to the aquaria, (2) Intermittent Predator: we added 100 ml of water taken from aquaria holding fed *Anax* (fed ~ 200 mg tadpoles per day) to the aquaria with tadpoles, (3) Constant Predator: we added one *Anax* to each nylon cage suspended within an aquarium and fed it two tadpoles (~ 200 mg) daily. The three predator treatments were crossed with three hormone manipulation treatments: vehicle control (0.001% ethanol; No Hormone treatment), 125 nM CORT, or 110 μM MTP. We chose this dose of CORT based on prior experiments to elevate whole-body CORT within the physiological range observed during predator exposure^28^. Likewise, we chose the dose of MTP based on prior experiments to maintain whole-body CORT at control levels during predator exposure^29^. We produced stock solutions of 125 μM CORT and 110 mM MTP by dissolving each in 100% ethanol. We applied CORT or MTP treatments by adding the appropriate volume of the stock solution to each aquarium using a pipette. Vehicle controls received ethanol to a final concentration of 0.001%. We applied the hormone manipulation treatments at the beginning of the experiment (Day 0); we then refreshed them every four days after a half-water change of all containers at 09:00 EDT. We placed *Anax* into the cages at the beginning of the experiment, then checked and fed them daily at 10:00 EDT. We added the water to the other two predator treatments at the beginning of the experiment and at the same time that the caged *Anax* were fed. No mortality of *Anax* or experimental tadpoles occurred during the experiment, and all caged *Anax* had consumed their prey tadpoles when checked each day.

On Days 0, 4, and 8 we haphazardly removed 9 tadpoles from each aquarium, leaving a tadpole density of 1 per 175 mL, 215 mL, and 275 mL, respectively, and an approximate biomass density of 850 mg/L, 700 mg/L, and 545 mg/L, respectively. We moved five of the tadpoles into new aquaria for predation trials (described below) and euthanized four. We weighed the four euthanized tadpoles and photographed them for morphological analysis, then flash froze them using a dry ice-ethanol slurry for direct measurement of whole-body CORT content. Photographs were taken by laying the tadpoles on their side on a white surface next to a measurement standard, straightening the tail, and photographing from ~ 30 cm directly above to reduce the barrel effect^30^ using a video camera with a 26.8 mm lens. Additionally, we selected four tadpoles from each container on Day 1 for a CORT measurement. Because of limited space in the lab room, we did not include tadpoles from the Intermittent Predator treatment in the predation trials, but did remove them from their treatment aquaria and measure them.

### Predation trials

We conducted predation trials (four replicates of six treatment combinations for 24 total trials each during Days 0, 4, and 8) in 20 × 34 × 12 cm aquaria filled with 3.5 L of aged, dechlorinated city water. We chose the size of the aquaria to be small enough for all locations to be within the visual range of *Anax*^31^ in order to minimize the role of behavioral defenses of tadpoles (i.e., spatial avoidance or activity reduction that reduces risk through reduction of encounter rate). This configuration allowed us to focus on the role of the tadpole morphological response in their defense during an attack by an *Anax*. We laid strips of fiberglass screen flat along the bottom of the container to provide structure. We marked each aquarium with a unique identification number, but all were otherwise identical. We allowed tadpoles to acclimate for one hour after being moved into the aquaria. All aquaria were recorded from directly above continuously for the duration of the trial. To begin the predation trials, we placed one middle-instar *Anax* dragonfly at the center of each aquarium. We used different *Anax* for each trial, and all had been held without feeding for 24 h prior to the trial. After 2 h, we counted all surviving tadpoles and inspected them for bodily damage.

### CORT extraction

We conducted organic extraction of whole tadpoles to analyze whole-body CORT content as described by Denver^32^ with modifications. Samples were combined within the same hormone manipulation treatments to confirm that treatments were applied effectively, and to increase sample volume and recovery. We pooled the four euthanized tadpoles from each treatment for each replicate before homogenizing them in ethyl acetate. We then added 3000 cpm of [^3^H]CORT to each sample to monitor recoveries, dried them in a Speedvac, then resuspended them in 100 μL dichloromethane:methanol (98:2). We fractionated the tadpole extracts using Sephadex LH-20 columns (Sigma-Aldrich, St. Louis, MO, USA) with a 20 ml bed volume. After applying samples to the columns, we added 20 mL of dichloromethane:methanol (98:2) and collected five 1 mL fractions that contained the CORT; we determined the CORT elution profile in a pilot experiment using [^3^H]CORT added to tadpole extract. We dried the fractions in a Speedvac, resuspended them in PBS with 1% gelatin and analyzed CORT concentration in the extracts using enzyme-linked immunoassay (Cayman Biochemicals, Ann Arbor, MI, USA) following the manufacturer’s instructions. Recoveries ranged from 40 to 62%.

### Data collection and analysis

From videos, two observers measured the activity of the tadpoles in each treatment combination by pausing the video for Days 0, 4, and 8 at 55 min after the tadpoles were added to the containers (i.e., 5 min before the *Anax* were added to begin the predation trials), then choosing five tadpoles from each replicate and recording the amount of time in seconds during 1 min that each tadpole was active (i.e., swimming or feeding). We averaged activity measurements from tadpoles housed in the same container between observers, and used the resulting replicate mean as the experimental unit (one activity level per replicate). We compared mean activity among treatments and through time using a linear mixed-effects model with treatment and day as fixed effects and replicate as a random effect using package lme4, version 1.1-23^33^ in R^34^, after confirming that assumptions of homogeneity of variance (Levene’s test) and a normal distribution of residuals (Q-Q plots) were met. We conducted post-hoc pairwise comparisons using package lsmeans, version 2.30-0^35^.

From the video of the predation trials, two independent observers recorded the timing and number of strikes made by *Anax* (i.e., attacks) and the outcome (tadpole escape or capture). Comparison of observations showed that both observers agreed on the timing of all strikes and the outcomes. We used two measures of tadpole performance. The first was *Anax* attack success rate, which should be less affected by differences in encounter rates due to tadpole or *Anax* behavior or production of chemical cues than other possible measures such as survival rate. We compared the counts of escapes and captures among the six treatment combinations in each trial using generalized linear models with binomial distributions and log link functions (R package lme4, version 1.1-23)^33^. The No Predator/CORT treatment combination was removed from the analysis for Day 4 because no strikes occurred in three of the replicates and only one unsuccessful strike occurred in the fourth. Multiple strikes occurred in all other replicates of other treatment combinations. The second measure of tadpole performance was time to first capture, which was assessed using an accelerated failure time model because of the possibility of behavioral responses changing predator encounter or attack rates (R package survival, version 3.1-8)^36^. In addition to these two metrics, the observers also sought to record the reaction distance of the tadpoles to the predator. However, while tadpoles occasionally swam away from an approaching *Anax* prior to attack, we observed little difference in reaction distance between successful and unsuccessful attacks (i.e., attacks which did or did not end in capture). We observed attacks only when *Anax* were able to approach to within ~ 1–2 cm of a tadpole, which was too short a distance to precisely measure given the frame rate we used.

We conducted morphological analysis of tadpoles (tail muscle and tail fin depth) using Image J 1.36b^37^ based on the landmarks defined by Relyea^38^. To remove effects of body size, we regressed trait measurements on total lengths using an analysis of covariance design^39^. We averaged size-corrected morphological measurements from tadpoles housed in the same container and used the resulting replicate mean as the experimental unit. We compared body weight and the mean mass-corrected tail muscle and tail fin depth among treatments and at different time points using a linear mixed-effects model with treatment and day as fixed effects and replicate as a random effect using package lme4^33^ in R^34^, after confirming that assumptions of homogeneity of variance (Levene’s test) and a normal distribution of residuals (Q-Q plots) were met. We conducted post-hoc pairwise comparisons using package lsmeans, version 2.30-0^35^.

We compared whole-body CORT content among the hormone manipulation treatments (No predator) using analysis of variance.

## Results

Our results show that CORT treatment was effective at elevating whole-body CORT content within the physiological range, while MTP treatment maintained whole-body CORT content at or below the control level during predator exposure. Treatment of tadpoles with exogenous CORT caused a statistically significant increase in whole-body CORT content relative to tadpoles treated with MTP or the ethanol vehicle after one day of exposure (ethanol vehicle control: 3903 ± 1286 s.e.m. pg CORT/mg body weight, CORT: 12,690 ± 4769, MTP: 1062 ± 189, *F*_2,26_ = 15.3, *P* = 0.001), and whole-body CORT content remained elevated through Day 8 (ethanol vehicle control: 904 ± 421 pg CORT/mg body weight, CORT: 2989 ± 497, MTP: 539 ± 65, *F*_2,26_ = 9.3, *P* = 0.007). No differences in whole-body CORT content were observed between MTP-treated and control tadpoles.

**Figure 1:**
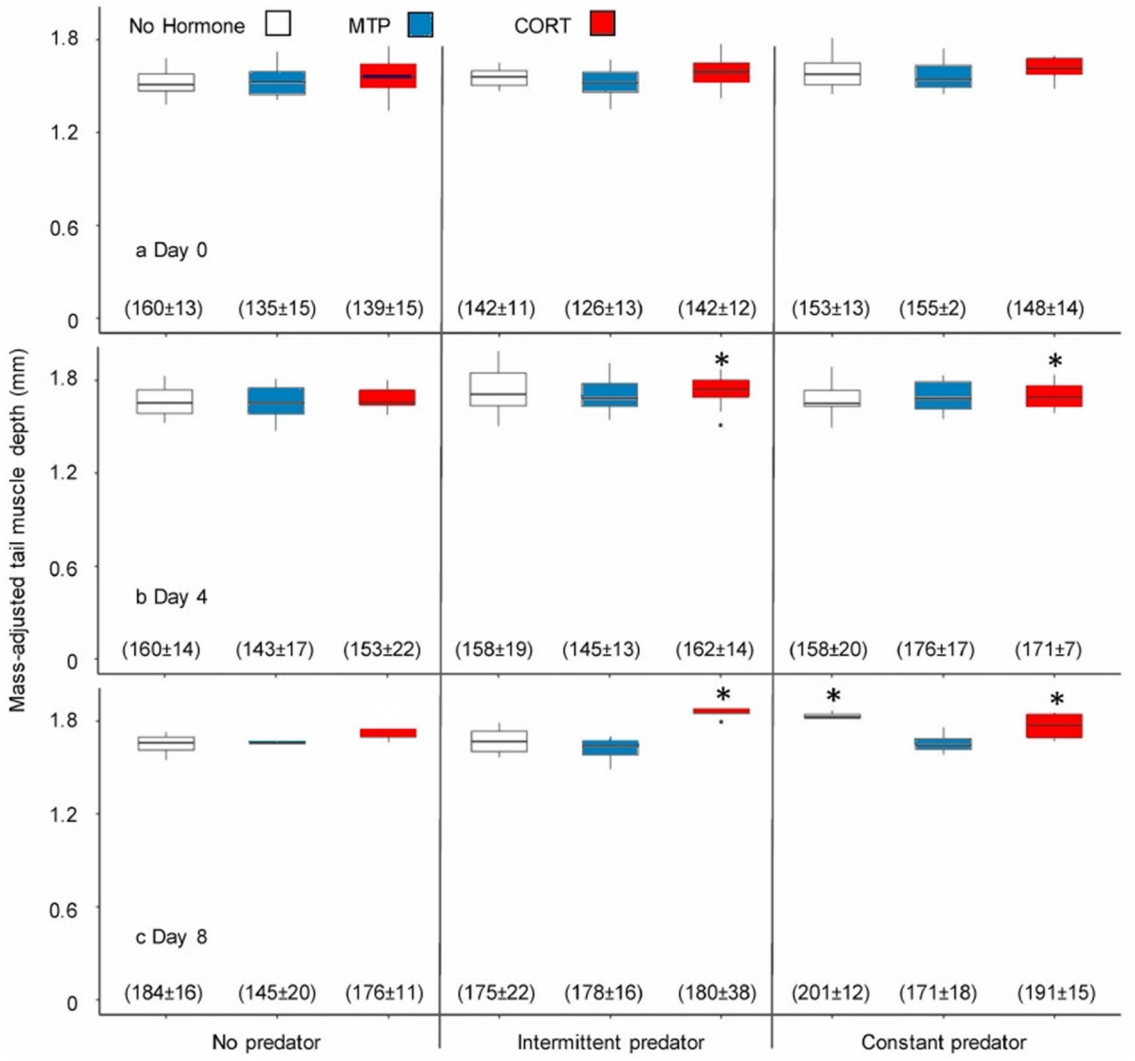
Treatment of wood frog tadpoles with corticosterone (CORT) or nonlethal exposure to a predator increased tail muscle depth, while treatment with the corticosteroid synthesis inhibitor metyrapone (MTP) blocked the tail muscle response to the predator. Shown are boxplots (median and interquartile range) of mass-adjusted tail muscle depth in each treatment combination (*n* = 4 replicates) on Day 0 (**a**), Day 4 (**b**), and Day 8 (**c**). Mean individual tadpole mass (± s.e.m.) is given below each box. Asterisks indicate statistically significant differences compared to the No predator/No hormone treatment (Bonferroni-corrected *P* < 0.05). Other statistically significant pairwise contrasts are given in Supplementary Table S1.

Tadpole body weight increased over time (*χ*_1_ = 27.5, *P* < 0.001, Fig. 1), and we did not observe statistically significant differences among treatments or a treatment x day interaction. Tadpoles exposed to *Anax* and exogenous CORT developed deeper tail muscles with time, whereas MTP blocked this response (Fig. 1). We observed statistically significant differences among treatments (*χ*_8_ = 42.7, *P* < 0.001) and a treatment x time interaction (*χ*_16_ = 28.7, *P* = 0.026). On Day 4, CORT plus constant or intermittent predator exposure resulted in increased tail muscle depth compared to the no predator-no hormone control (Fig. 1b, Supplementary Table S1). On Day 8, CORT plus constant or intermittent predator exposure again resulted in increased tail muscle depth versus the control, as did constant predator exposure alone (Fig. 1c, Supplementary Table S1). Intermittent predator exposure did not affect tail muscle depth versus the control at any time point. However, CORT plus intermittent predator exposure strongly increased tail muscle depth (Supplementary Table S1), indicating a synergy. The effect of predator exposure was completely blocked by MTP, and MTP alone had no effect on tail muscle depth versus the control at any time point. Tail fin depth exhibited some similar patterns to tail muscle depth, with significantly deeper tail fins observed on Day 8 among all constant predator exposure treatments and the intermittent predator exposure plus CORT treatment compared to the No predator-No hormone control (Fig. S1). However, the tadpoles in the No predator-MTP and Constant predator-MTP treatments also unexpectedly exhibited significantly deeper tail fins on Day 8 (Fig. S1).

**Figure 2:**
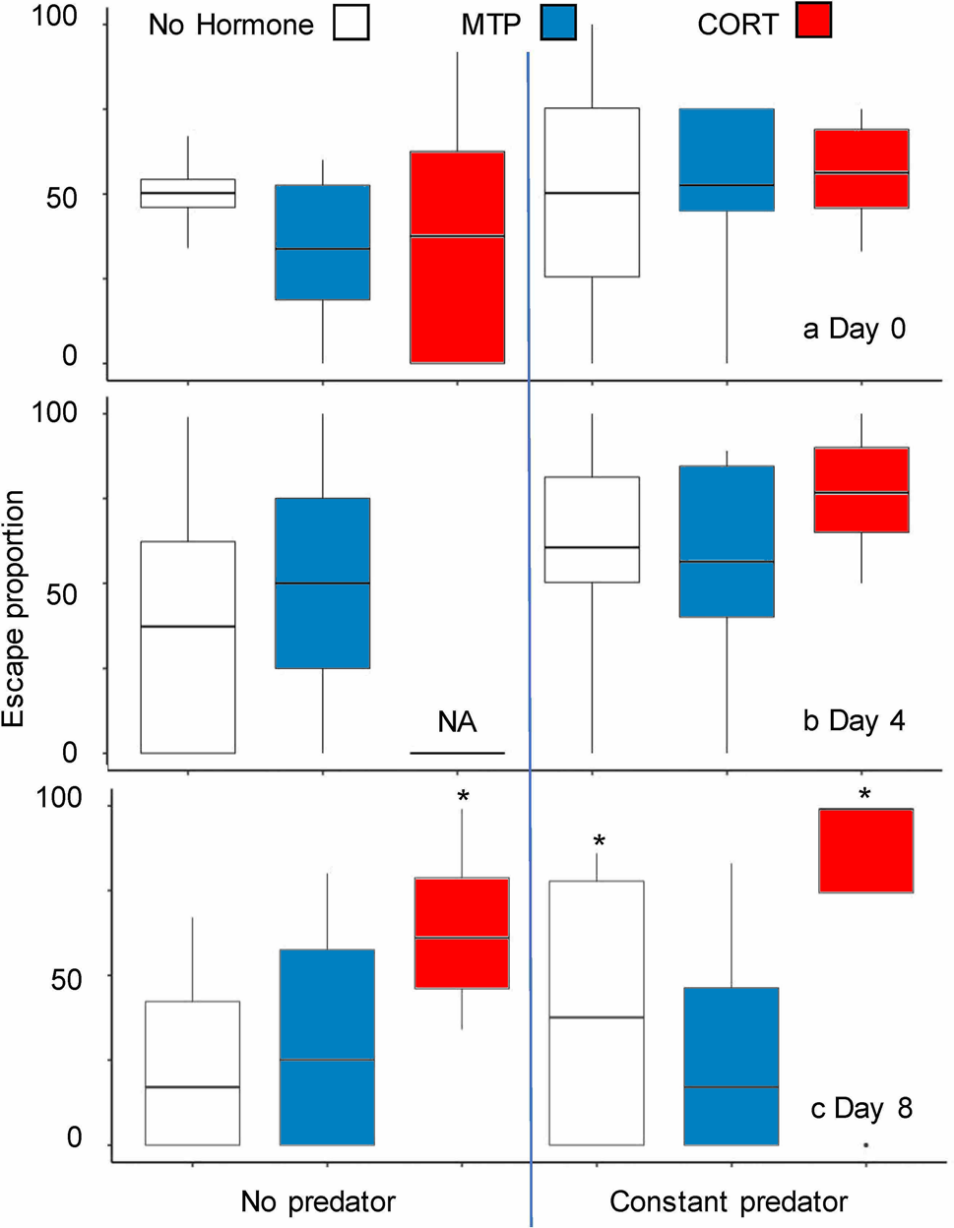
Treatment with corticosterone (CORT) or constant exposure to nonlethal predators increased the ability of wood frog tadpoles to escape predator attack. Shown are boxplots (median and interquartile range) of percentage of *Anax* attacks from which tadpoles successfully escaped in each treatment combination (*n* = 4 replicates) on Day 0 (**a**), Day 4 (**b**), and Day 8 (**c**). NA indicates a treatment in which no data were recorded due to technical problems. Asterisks indicate significant differences compared to the No predator/No hormone treatment (*P* < 0.05).

Escape performance (Fig. 2) and time to first capture also increased with time in tadpoles exposed to *Anax* and (or) exogenous CORT, with MTP again blocking these responses. Unlike the morphological results, no trends in *Anax* attack success were apparent on Day 4 (χ_5_ = 6.6, *P* = 0.3), and statistically significant differences were first detected on Day 8 (χ_5_ = 13.5, *P* = 0.006). On Day 8, tadpole escape success was higher (i.e., *Anax* attack success was lower) for Constant Predator/No Hormone, No Predator/CORT, and Constant Predator/CORT treatments compared to the No Predator/No Hormone treatment (Fig. 2c). An accelerated failure time model indicated no significant differences among treatments in time to first capture on Day 0 (*C*_5_ = 1.1, *P* = 0.9) or Day 4 (*C*_5_ = 1.1, *P* = 0.9). On Day 8, a statistically significant difference was found (*C*_5_ = 15.5, *P* = 0.008), which was driven by increased survival durations in the Constant Predator/CORT (mean ± SE time to first capture, 3200 ± 1500 s), Constant Predator/No Hormone (2000 ± 1600 s), and No Predator/CORT treatments (2100 ± 1400 s) relative to the No Predator/No Hormone treatment (400 ± 100 s). Additionally, we observed no damage to the tails of surviving tadpoles at the end of the predation trials in this experiment (although some *Anax* did capture tadpoles with strikes to their tails, MF pers. obs.).

**Figure 3:**
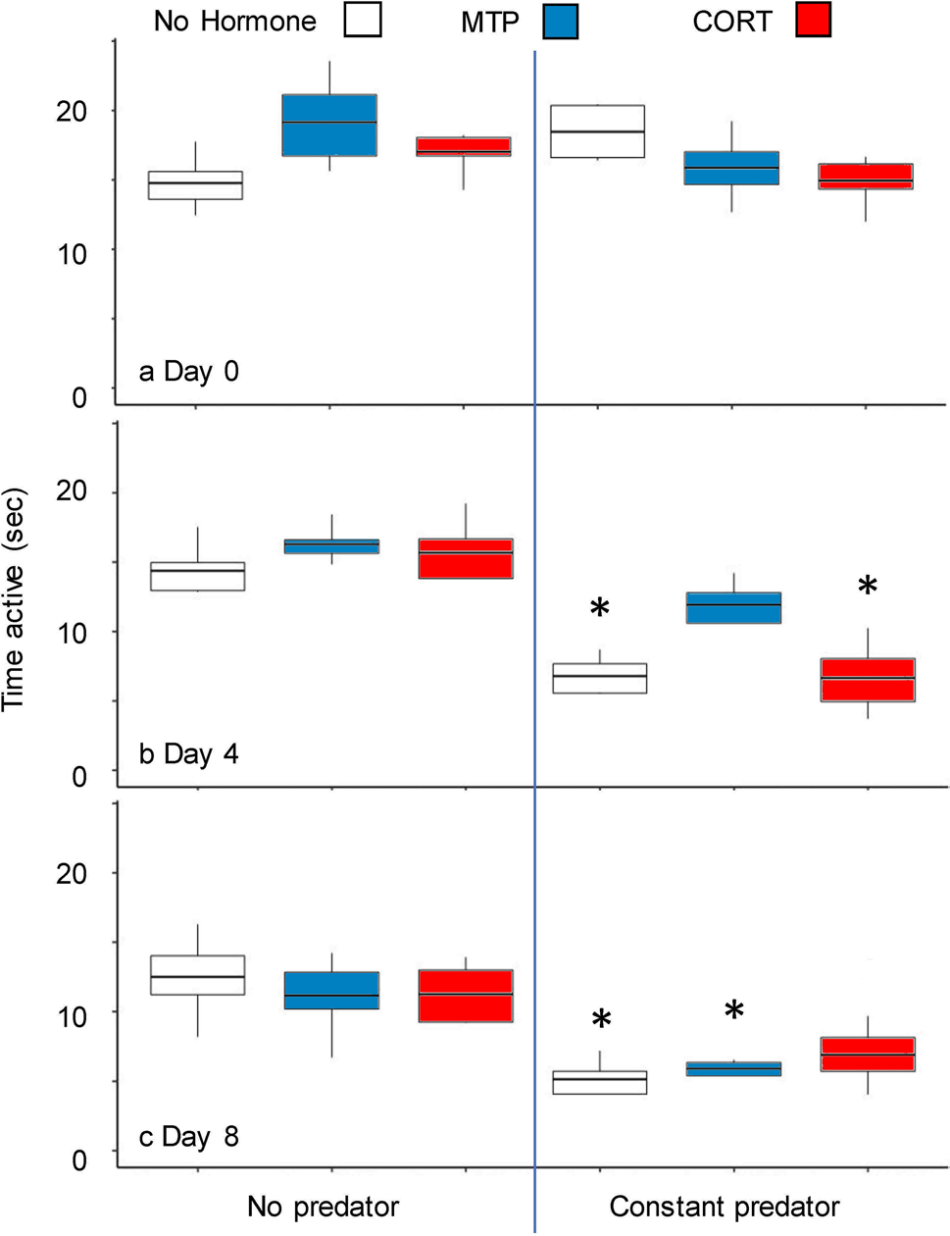
Treatment of wood frog tadpoles with nonlethal exposure to a predator resulted in decreased activity. Shown are boxplots (median and interquartile range) of swimming activity of tadpoles in each treatment combination (*n* = 4 replicates) on Day 0 (**a**), Day 4 (**b**), and Day 8 (**c**). Asterisks indicate significant differences compared to the No predator/No hormone treatment (Bonferroni-corrected *P* < 0.05). Other significant pairwise contrasts are noted in Supplementary Table S2.

Tadpole activity levels tended to decrease in response to *Anax* exposure and remain lower than the No Predator treatments through Day 8 (Fig. 3). Activity was visually lower in *Anax* treatments on Day 1, although not recorded on video (MF, pers. obs.). We observed significant differences among treatments (*X*_5_ = 69.2, *P* < 0.001) and days (*X*_*2*_ = 6.6, *P* = 0.036) and a significant treatment x day interaction (*X*_10_ = 46.8, *P* < 0.001). On Day 4, activity levels in the Constant Predator/No Hormone and Constant Predator/CORT treatments were lower than in the No Predator/No Hormone treatment (Fig. 3b, Supplementary Table S2), but activity level in the Constant Predator/MTP treatment was not. On Day 8, activity levels in the Constant Predator/No Hormone and Constant Predator/MTP treatments were lower than in the No Predator/No Hormone treatment, but were not in the Constant Predator/CORT treatment (Fig. 3c, Supplementary Table S2).

## Discussion

We showed that wood frog tadpoles exposed to caged *Anax* alone or with exogenous CORT developed deeper tail muscles and exhibited improved probability of escaping *Anax* attacks after 4 to 8 days of exposure. Application of MTP blocked both this response and the improved escape performance. These results further link the predator-induced physiological stress response in wood frog tadpoles^7,16^ to the expression of an antipredator morphology and to an adaptive benefit in survival probability mediated by improved escape performance.

The tail morphological response was consistent with our expectations and previous studies^7^. The lack of observed tail damage to surviving tadpoles after the predation trials suggests that the improved escape performance was more consistent with improved escape swimming rather than a lure effect, although we did not measure burst swimming speed and the lure effect could also operate by causing more predator strikes to miss completely. The full induced tail morphology (muscle and overall fin depth) may improve survival through several interacting mechanisms; for example, improved escape speed could help deflect attacks aimed at the tail^40^ or result in predator strikes missing completely. Observations of tadpoles across several species in nature suggest that survival is possible with some degree of tail damage^41^. It is also possible that predator- and CORT-exposed tadpoles became more vigilant or responsive in a way that improved their escape performance. While the induced tail morphology is widely observed and likely adaptive, more focused studies on the development of the overall induced morphology and its effect on predator-tadpole interaction will be necessary to disentangle the mechanisms of how it enhances survival.

Our results further clarify the role of stress hormones in the development of the antipredator response, but also point to the need to describe the dose–response relationship. Consistent with the role of stress hormones, the morphological patterns also suggest that antipredator morphological development is influenced by the level of predation risk. Tadpoles that experienced high predation risk or artificially elevated stress hormone levels expressed morphological responses, whereas those that experienced lower stress (controls or MTP treated tadpoles) did not. For example, tadpoles in the Constant Predator treatments and in treatments combining Constant or Intermittent Predator exposure and CORT exhibited a morphological response on Day 8, whereas tadpoles in the Intermittent Predator/No Hormone and No Predator/CORT treatment combinations did not, although they trended in that direction. Tadpoles have previously shown risk-sensitive stress responses to predator density and chemical cue concentration^7,16^. Clarifying how the environment, including patterns and levels of predation risk, types of and variation in information sources, resource levels, and time for development, influences the stress response is necessary to fully describe how it regulates the expression of plastic traits in nature^4^. Future experiments should quantify whole-body CORT content at a finer temporal scale and more closely link it to changes in morphology or other phenotypic characteristics to identify the shapes of relationships (e.g., linear, nonlinear). Additionally, following responses and performance of individual tadpoles rather than using group means would improve the precision of future experiments by testing for direct links from an individual’s stress response to its phenotype and performance. Following individual tadpoles or small groups would also allow experiments to be conducted using lower densities than those used here, which potentially could have produced a confounding stress response (although, note that our present results are in line with prior results conducted at lower densities^7,16^). Previous studies have found that conspecific density can influence both risk assessment^42^ and the phenotypic response to predation risk^29,43^. Further work on how density and other stressors interact with predator stress-induced defenses could be informative, as prey in the field are frequently exposed to multiple stressors that can vary over time or space.

Large increases in escape success suggest that antipredator morphology can be an effective defense in tadpoles. This is consistent with previous studies that have found morphological defenses contribute to reduced predation vulnerability, although how morphological defenses interact with other defensive traits remains less understood^27,44^. However, the time needed for development (4–8 days in this study, with similar rates found in other studies on wood frogs^7^ and heterospecifics^45^) and the possible allocation costs (e.g., to other growth or development) may offset some of the benefit. Additionally, the tail morphology acts at the attack portion of the predation sequence, whereas other defenses (e.g., activity level) may act earlier to reduce predator encounter rates. As with most taxa, tadpoles exhibit multiple defensive traits^19,46^. How these traits are integrated determines the overall adaptive value of the phenotype^46,47^. Behavioral responses, owing to their rapid expression, can complement more slowly developing morphological responses to reduce encounter rates with predators, and other plastic traits (e.g., metabolism)^48^ may help to reduce costs of behavioral responses (e.g., lost foraging opportunities). Previous studies suggest that the glucocorticoid signaling pathway also is involved in the expression of antipredator behavior in tadpoles; wood frog tadpoles initially exposed to chemical cues exhibit a short-term decrease in whole-body CORT that permits an activity reduction^16^. Better understanding of the assessment-outcome sequence for behavior and how behavior and morphology are integrated into an overall defense^46^ through time would be useful. Such understanding could help explain how tadpoles reduce risk both during the morphological development period and over their full larval stage. Understanding the sequences may also help identify any tradeoffs involved, and suggest when integrating defenses is favorable and when the optimal response may be to allocate to one defense instead (e.g., only express behavioral defenses).

While the activity reduction in response to predator exposure that we observed is consistent with some longer-term experiments^27,46^, it is somewhat inconsistent with our previous finding that tadpoles treated with exogenous CORT during predator exposure resumed control levels of activity sooner than those that were not^16^. This disparity may reflect a difference in responses between acute, discrete periods of risk and chronic risk. As with the tail morphological response, better quantification of whole-body CORT content at finer temporal scales and linking the stress response to environmental variation and phenotypic responses is needed. Additionally, defensive behavior not considered here (e.g., space use) may be part of the overall antipredator phenotype and is likely to also be regulated by the stress response. Finally, further work linking information sources (e.g., risk cues^49^; as well as safety cues^50^) to the stress response will help to clarify the induction sequence.

Describing the induction sequences that link environmental conditions to physiological responses to phenotypic expression to individual performance will improve our understanding of the mechanisms underlying many predator–prey interactions^3,4^. Antipredator responses can consist of a suite of traits (e.g., behavioral, morphological, and developmental traits that can directly influence the interaction, as well as more compensatory traits such as metabolism^51^) that act at different points in the predation sequence (i.e., encounter, attack, capture). Therefore, understanding the integration of antipredator traits will lead to the ultimate goal to understanding how predator-induced phenotypic plasticity influences ecosystems^47^. However, a key initial step is to develop a functional description of the induction sequence for particular traits. This step will help to clarify how plastic responses develop and operate, where tradeoffs or other interactions with other traits may occur, and what their potential impacts are.

## Supplementary Information

Supplementary Information.

## Data Availability

The data used in our analysis will be made available upon reasonable request to the corresponding author.

## References

[1] Tollrian R, Harvell CD (1998). The Ecology and Evolution of Inducible Defenses.

[2] Ohgushi T, Schmitz OJ, Holt RD (2013). Trait-Mediated Indirect Interactions: Ecological and Evolutionary Perspectives.

[3] Ellers J, Stuefer JF (2010). Frontiers in phenotypic plasticity research: new questions about mechanisms, induced responses, and ecological impacts. Evol. Ecol..

[4] Mitchell MD, Bairos-Novak KR, Ferrari MC (2017). Mechanisms underlying the control of responses to predator odours in aquatic prey. J. Exp. Biol..

[5] Stankowich T, Blumstein DT (2005). Fear in animals: a meta-analysis and review of risk assessment. Proc. Roy. Soc. B Biol. Sci..

[6] Brönmark C, Hansson L-A (2012). Chemical Ecology in Aquatic Systems.

[7] Middlemis Maher J, Werner EE, Denver RJ (2013). Stress hormones mediate predator-induced phenotypic plasticity in amphibian tadpoles. Proc. R. Soc. B Biol. Sci..

[8] Dennis SR, LeBlanc GA, Beckerman AP (2014). Endocrine regulation of predator-induced phenotypic plasticity. Oecologia.

[9] Matsunami M (2015). Transcriptome analysis of predator- and prey-induced phenotypic plasticity in the Hokkaido salamander (*Hynobius retardatus*). Mol. Ecol..

[10] Weiss LC (2019). Sensory ecology of predator-induced phenotypic plasticity. Front. Behav. Neurosci..

[11] Hawlena D, Schmitz OJ (2010). Physiological stress as a fundamental mechanism linking predation to ecosystem functioning. Am. Nat..

[12] Auld JR, Relyea RA (2011). Adaptive plasticity in predator-induced defenses in a common freshwater snail: altered selection and mode of predation due to prey phenotype. Evol. Ecol..

[13] Meuthen D, Baldauf SA, Bakker TC, Thünken T (2018). Neglected patterns of variation in phenotypic plasticity: age-and sex-specific antipredator plasticity in a cichlid fish. Am. Nat..

[14] Schoeppner NM, Relyea RA (2009). Interpreting the smells of predation: how alarm cues and kairomones induce different prey defenses. Func. Ecol..

[15] Hettyey A (2015). The relative importance of prey-borne and predator-borne chemical cues for inducible antipredator responses in tadpoles. Oecologia.

[16] Fraker ME (2009). Characterization of an alarm pheromone secreted by amphibian tadpoles that induces behavioral inhibition and suppression of the neuroendocrine stress axis. Horm. Behav..

[17] Hossie TJ, Ferland-Raymond B, Burness G, Murray DL (2010). Morphological and behavioural responses of frog tadpoles to perceived predation risk: a possible role for corticosterone mediation?. Écoscience.

[18] McDiarmid RW, Altig R (1999). Tadpoles: the Biology of Anuran Larvae.

[19] Relyea RA (2004). Fine-tuned phenotypes: tadpole plasticity under 16 combinations of predators and competitors. Ecology.

[20] Wilson RS, Kraft PG, Van Damme R (2005). Predator-specific changes in the morphology and swimming performance of larval *Rana lessonae*. Func. Ecol..

[21] Van Buskirk J, McCollum SA (2000). Influence of tail shape on tadpole swimming performance. J. Exp. Biol..

[22] Eidietis L (2005). Size-related performance variation in the wood frog (*Rana sylvatica*) tadpole tactile-stimulated startle response. Can. J. Zool..

[23] Perotti MG, Pueta M, Jara FG, Úbeda CA, Moreno Azocar DL (2016). Lack of functional link in the tadpole morphology induced by predators. Curr. Zool..

[24] Mori T (2017). The constant threat from a non-native predator increases tail muscle and fast-start swimming performance in *Xenopus* tadpoles. Biol. Open.

[25] Lindgren B, Orizaola G, Laurila A (2018). Interacting effects of predation risk and resource level on escape speed of amphibian larvae along a latitudinal gradient. J. Evol. Biol..

[26] Van Buskirk J, Anderwald P, Lüpold S, Reinhardt L, Schuler H (2003). The lure effect, tadpole tail shape, and the target of dragonfly strikes. J. Herp..

[27] Dijk B, Laurila A, Orizaola G, Johansson F (2016). Is one defence enough? Disentangling the relative importance of morphological and behavioural predator-induced defences. Behav. Ecol. Sociobiol..

[28] Glennemeier KA, Denver RJ (2002). Moderate elevation of corticosterone content affects fitness components in northern leopard frog (*Rana pipiens*) tadpoles. Gen. Comp. Endocrinol..

[29] Glennemeier KA, Denver RJ (2002). Role for corticoids in mediating the response of *Rana pipiens* tadpoles to intraspecific competition. J. Exp. Zool..

[30] Muir AM, Vecsei P, Krueger CC (2012). A perspective on perspectives: methods to reduce variation in shape analysis of digital images. Trans. Am. Fish. Soc..

[31] Fraker ME, Luttbeg B (2012). Predator-prey space use and the spatial distribution of predation events. Behaviour.

[32] Denver RJ (1998). Hormonal correlates of environmentally induced metamorphosis in the western spadefoot toad, *Scaphiopus hammondii*. Gen. Comp. Endocrinol..

[33] Bates D, Mächler M, Bolker B, Walker S (2015). Fitting linear mixed-effects models using lme4. J. Stat. Soft..

[34] R Core Team. R: A language and environment for statistical computing, version 3.6.1. (R Foundation for Statistical Computing, 2019).

[35] Lenth RV (2016). Least-squares means: the R package lsmeans. J. Stat. Soft..

[36] Therneau, T. M. & Lumley, T. R Package ‘survival’ version 3.1-8 (2019).

[37] Schneider CA, Rasband WS, Eliceiri KW (2012). NIH Image to ImageJ: 25 years of image analysis. Nat. Methods.

[38] Relyea RA (2001). Morphological and behavioral plasticity of larval anurans in response to different predators. Ecology.

[39] Berner D (2011). Size correction in biology: how reliable are approaches based on (common) principal component analysis?. Oecologia.

[40] Humphreys RK, Ruxton GD (2018). What is known and what is not yet known about deflection of the point of a predator’s attack. Biol. J. Linn. Soc..

[41] Blair J, Wassersug RJ (2000). Variation in the pattern of predator-induced damage to tadpole tails. Copeia.

[42] Van Buskirk J, Ferrari M, Kueng D, Näpflin K, Ritter N (2011). Prey risk assessment depends on conspecific density. Oikos.

[43] McCoy MW (2007). Conspecific density determines the magnitude and character of predator-induced phenotype. Oecologia.

[44] Van Buskirk J, McCollum SA (2000). Functional mechanisms of an inducible defence in tadpoles: morphology and behaviour influence mortality risk from predation. J. Evol. Biol.

[45] Van Buskirk J (2002). Phenotypic lability and the evolution of predator-induced plasticity in tadpoles. Evolution.

[46] Hossie T, Landolt K, Murray DL (2017). Determinants and co-expression of anti-predator responses in amphibian tadpoles: a meta-analysis. Oikos.

[47] Laughlin DC, Messier J (2015). Fitness of multidimensional phenotypes in dynamic adaptive landscapes. Trends Ecol. Evol..

[48] Steiner UK, Van Buskirk J (2009). Predator-induced changes in metabolism cannot explain the growth/predation risk tradeoff. PLoS ONE.

[49] Ferrari MC, Wisenden BD, Chivers DP (2010). Chemical ecology of predator–prey interactions in aquatic ecosystems: a review and prospectus. Can. J. Zool..

[50] Luttbeg B, Ferrari MC, Blumstein DT, Chivers DP (2020). Safety cues can give prey more valuable information than danger cues. Am. Nat..

[51] Schmitz OJ (2017). Predator and prey functional traits: understanding the adaptive machinery driving predator–prey interactions. F1000Research.

