# Supplementary material for: Stress hormone-mediated antipredator morphology improves escape performance in amphibian tadpoles
Source: Sci Rep. 2021 Feb 24;11:4427. doi: 10.1038/s41598-021-84052-9 (PMC7904905; doi:10.1038/s41598-021-84052-9)
Supplement: Supplementary file 1 — Supplementary Information. [file 41598_2021_84052_MOESM1_ESM.pdf]

Stress hormone-mediated antipredator morphology improves escape performance in amphibian tadpoles

Michael E. Fraker, Stuart A. Ludsin, Barney Luttbeg, and Robert J. Denver

Supplementary Information

Supplementary Table S1. Pairwise contrasts among treatments within each day for tail muscle depth. Rows with an uncorrected *P* value < 0.05 are marked in bold. The rightside column indicates significance under the Bonferroni-corrected alpha (S: significant, NS: non-significant).

Supplementary Table S2. Pairwise contrasts among treatments within each day for swimming activity. Rows with an uncorrected *P* value < 0.05 are marked in bold. The rightside column indicates significance under the Bonferroni-corrected alpha (S: significant, NS: non-significant).

Supplementary Figure S1. Boxplots (median and interquartile range) of mass-adjusted tail depth of wood frog tadpoles in each treatment combination ($n = 4$ replicates) on Day 0 (a), Day 4 (b), and Day 8 (c). Asterisks indicate statistically significant differences compared to the No predator/No hormone treatment (Bonferroni-corrected $P < 0.05$).

Supplementary Table S1. Pairwise contrasts for tail muscle height among treatments during each day of observation.

Rows with an uncorrected P value < 0.05 are marked in bold. The rightside column indicates significance under the Bonferroni-corrected alpha (S: significant, NS: non-significant).

Day 0						
Contrast	Estimate	SE	df	t ratio	*P*	(Bonferroni alpha = 0.0014)
Con pred/CORT - Con pred/MTP	0.00894	0.0188	78	0.474	0.9999	NS
Con pred/CORT - Con pred/No horm	0.01711	0.0188	78	0.908	0.9919	NS
Con pred/CORT - Int pred/CORT	-0.02529	0.0188	78	-1.342	0.9152	NS
Con pred/CORT - Int pred/MTP	-0.01196	0.0188	78	-0.634	0.9993	NS
Con pred/CORT - Int pred/No horm	-0.02181	0.0188	78	-1.157	0.9628	NS
Con pred/CORT - No pred/CORT	0.01857	0.0188	78	0.986	0.9862	NS
Con pred/CORT - No pred/MTP	0.03615	0.0188	78	1.918	0.6034	NS
Con pred/CORT - No pred/No horm	0.03049	0.0188	78	1.618	0.7923	NS
Con pred/MTP - Con pred/No horm	0.00818	0.0188	78	0.434	1	NS
Con pred/MTP - Int pred/CORT	-0.03423	0.0188	78	-1.816	0.6715	NS
Con pred/MTP - Int pred/MTP	-0.02089	0.0188	78	-1.109	0.9713	NS
Con pred/MTP - Int pred/No horm	-0.03075	0.0188	78	-1.632	0.7845	NS
Con pred/MTP - No pred/CORT	0.00964	0.0188	78	0.511	0.9999	NS
Con pred/MTP - No pred/MTP	0.02722	0.0188	78	1.444	0.8768	NS
Con pred/MTP - No pred/No horm	0.02155	0.0188	78	1.143	0.9654	NS
Con pred/No horm - Int pred/CORT	-0.0424	0.0188	78	-2.25	0.385	NS
Con pred/No horm - Int pred/MTP	-0.02907	0.0188	78	-1.542	0.8318	NS
Con pred/No horm - Int pred/No horm	-0.03893	0.0188	78	-2.065	0.504	NS
Con pred/No horm - No pred/CORT	0.00146	0.0188	78	0.078	1	NS
Con pred/No horm - No pred/MTP	0.01904	0.0188	78	1.01	0.9838	NS
Con pred/No horm - No pred/No horm	0.01337	0.0188	78	0.71	0.9985	NS
Int pred/CORT - Int pred/MTP	0.01333	0.0188	78	0.708	0.9986	NS
Int pred/CORT - Int pred/No horm	0.00348	0.0188	78	0.185	1	NS
Int pred/CORT - No pred/CORT	0.04387	0.0188	78	2.328	0.3389	NS
**Int pred/CORT - No pred/MTP**	**0.06145**	**0.0188**	**78**	**3.26**	**0.0414**	**NS**
Int pred/CORT - No pred/No horm	0.05578	0.0188	78	2.96	0.0905	NS
Int pred/MTP - Int pred/No horm	-0.00986	0.0188	78	-0.523	0.9998	NS
Int pred/MTP - No pred/CORT	0.03053	0.0188	78	1.62	0.791	NS
Int pred/MTP - No pred/MTP	0.04811	0.0188	78	2.553	0.2239	NS
Int pred/MTP - No pred/No horm	0.04244	0.0188	78	2.252	0.3837	NS
Int pred/No horm - No pred/CORT	0.04039	0.0188	78	2.143	0.4526	NS
Int pred/No horm - No pred/MTP	0.05797	0.0188	78	3.076	0.0676	NS
Int pred/No horm - No pred/No horm	0.0523	0.0188	78	2.775	0.1398	NS

No pred/CORT - No pred/MTP	0.01758	0.0188	78	0.933	0.9903	NS
No pred/CORT - No pred/No horm	0.01191	0.0188	78	0.632	0.9994	NS
No pred/MTP - No pred/No horm	-0.00567	0.0188	78	-0.301	1	NS
Day 4						
Contrast	Estimate	SE	df	t ratio	*P*	(Bonferroni alpha = 0.0014)
**Con pred/CORT - Con pred/MTP**	**0.0779**	**0.0188**	**78**	**4.134**	**0.0027**	**NS**
**Con pred/CORT - Con pred/No horm**	**0.06368**	**0.0188**	**78**	**3.379**	**0.0297**	**NS**
Con pred/CORT - Int pred/CORT	0.03148	0.0188	78	1.67	0.7624	NS
**Con pred/CORT - Int pred/MTP**	**0.11869**	**0.0188**	**78**	**6.298**	**<.0001**	**S**
**Con pred/CORT - Int pred/No horm**	**0.08756**	**0.0188**	**78**	**4.646**	**0.0004**	**S**
**Con pred/CORT - No pred/CORT**	**0.06985**	**0.0188**	**78**	**3.706**	**0.0111**	**NS**
**Con pred/CORT - No pred/MTP**	**0.10731**	**0.0188**	**78**	**5.694**	**<0.0001**	**S**
**Con pred/CORT - No pred/No horm**	**0.1156**	**0.0188**	**78**	**6.134**	**<0.0001**	**S**
Con pred/MTP - Con pred/No horm	-0.01422	0.0188	78	-0.755	0.9977	NS
Con pred/MTP - Int pred/CORT	-0.04643	0.0188	78	-2.463	0.266	NS
Con pred/MTP - Int pred/MTP	0.04079	0.0188	78	2.164	0.4389	NS
Con pred/MTP - Int pred/No horm	0.00966	0.0188	78	0.513	0.9999	NS
Con pred/MTP - No pred/CORT	-0.00806	0.0188	78	-0.427	1	NS
Con pred/MTP - No pred/MTP	0.02941	0.0188	78	1.56	0.8227	NS
Con pred/MTP - No pred/No horm	0.0377	0.0188	78	2	0.5478	NS
Con pred/No horm - Int pred/CORT	-0.03221	0.0188	78	-1.709	0.7393	NS
Con pred/No horm - Int pred/MTP	0.05501	0.0188	78	2.919	0.1	NS
Con pred/No horm - Int pred/No horm	0.02388	0.0188	78	1.267	0.9378	NS
Con pred/No horm - No pred/CORT	0.00616	0.0188	78	0.327	1	NS
Con pred/No horm - No pred/MTP	0.04363	0.0188	78	2.315	0.3462	NS
Con pred/No horm - No pred/No horm	0.05192	0.0188	78	2.755	0.1462	NS
**Int pred/CORT - Int pred/MTP**	**0.08721**	**0.0188**	**78**	**4.628**	**0.0005**	**S**
**Int pred/CORT - Int pred/No horm**	**0.05609**	**0.0188**	**78**	**2.976**	**0.087**	**NS**
Int pred/CORT - No pred/CORT	0.03837	0.0188	78	2.036	0.5238	NS
**Int pred/CORT - No pred/MTP**	**0.07584**	**0.0188**	**78**	**4.024**	**0.004**	**NS**
**Int pred/CORT - No pred/No horm**	**0.08413**	**0.0188**	**78**	**4.464**	**0.0009**	**S**
Int pred/MTP - Int pred/No horm	-0.03113	0.0188	78	-1.652	0.7732	NS
Int pred/MTP - No pred/CORT	-0.04884	0.0188	78	-2.592	0.2071	NS
Int pred/MTP - No pred/MTP	-0.01138	0.0188	78	-0.604	0.9995	NS
Int pred/MTP - No pred/No horm	-0.00309	0.0188	78	-0.164	1	NS
Int pred/No horm - No pred/CORT	-0.01772	0.0188	78	-0.94	0.9898	NS

Int pred/No horm - No pred/MTP	0.01975	0.0188	78	1.048	0.9796	NS	
Int pred/No horm - No pred/No horm	0.02804	0.0188	78	1.488	0.8578	NS	
No pred/CORT - No pred/MTP	0.03747	0.0188	78	1.988	0.5563	NS	
No pred/CORT - No pred/No horm	0.04576	0.0188	78	2.428	0.284	NS	
No pred/MTP - No pred/No horm	0.00829	0.0188	78	0.44	1	NS	
Day 8							
Contrast	Estimate	SE	df	t ratio	*P*	(Bonferroni-corrected alpha = 0.0014)	
**Con pred/CORT - Con pred/MTP**	**0.09012**	**0.0188**	**78**	**4.782**	**0.0003**	**S**	
Con pred/CORT - Con pred/No horm	-0.05799	0.0188	78	-3.077	0.0674	NS	
**Con pred/CORT - Int pred/CORT**	**-0.07513**	**0.0188**	**78**	**-3.987**	**0.0045**	**NS**	
**Con pred/CORT - Int pred/MTP**	**0.12507**	**0.0188**	**78**	**6.636**	**<0.0001**	**S**	
**Con pred/CORT - Int pred/No horm**	**0.07836**	**0.0188**	**78**	**4.158**	**0.0025**	**NS**	
Con pred/CORT - No pred/CORT	0.02754	0.0188	78	1.461	0.8696	NS	
**Con pred/CORT - No pred/MTP**	**0.089**	**0.0188**	**78**	**4.722**	**0.0003**	**S**	
**Con pred/CORT - No pred/No horm**	**0.09738**	**0.0188**	**78**	**5.167**	**0.0001**	**S**	
**Con pred/MTP - Con pred/No horm**	**-0.14811**	**0.0188**	**78**	**-7.859**	**<0.0001**	**S**	
**Con pred/MTP - Int pred/CORT**	**-0.16525**	**0.0188**	**78**	**-8.768**	**<0.0001**	**S**	
Con pred/MTP - Int pred/MTP	0.03495	0.0188	78	1.854	0.6464	NS	
Con pred/MTP - Int pred/No horm	-0.01176	0.0188	78	-0.624	0.9994	NS	
**Con pred/MTP - No pred/CORT**	**-0.06258**	**0.0188**	**78**	**-3.32**	**0.035**	**NS**	
Con pred/MTP - No pred/MTP	-0.00112	0.0188	78	-0.059	1	NS	
Con pred/MTP - No pred/No horm	0.00726	0.0188	78	0.385	1	NS	
Con pred/No horm - Int pred/CORT	-0.01714	0.0188	78	-0.91	0.9918	NS	
**Con pred/No horm - Int pred/MTP**	**0.18306**	**0.0188**	**78**	**9.713**	**<0.0001**	**S**	
**Con pred/No horm - Int pred/No horm**	**0.13635**	**0.0188**	**78**	**7.235**	**<0.0001**	**S**	
**Con pred/No horm - No pred/CORT**	**0.08553**	**0.0188**	**78**	**4.538**	**0.0007**	**S**	
**Con pred/No horm - No pred/MTP**	**0.14699**	**0.0188**	**78**	**7.799**	**<0.0001**	**S**	
**Con pred/No horm - No pred/No horm**	**0.15537**	**0.0188**	**78**	**8.244**	**<0.0001**	**S**	
**Int pred/CORT - Int pred/MTP**	**0.2002**	**0.0188**	**78**	**10.623**	**<0.0001**	**S**	
**Int pred/CORT - Int pred/No horm**	**0.1535**	**0.0188**	**78**	**8.145**	**<0.0001**	**S**	
**Int pred/CORT - No pred/CORT**	**0.10267**	**0.0188**	**78**	**5.448**	**<0.0001**	**S**	
**Int pred/CORT - No pred/MTP**	**0.16413**	**0.0188**	**78**	**8.709**	**<0.0001**	**S**	
**Int pred/CORT - No pred/No horm**	**0.17251**	**0.0188**	**78**	**9.154**	**<0.0001**	**S**	
Int pred/MTP - Int pred/No horm	-0.0467	0.0188	78	-2.478	0.2588	NS	
**Int pred/MTP - No pred/CORT**	**-0.09753**	**0.0188**	**78**	**-5.175**	**0.0001**	**S**	
Int pred/MTP - No pred/MTP	-0.03607	0.0188	78	-1.914	0.6066	NS	

Int pred/MTP - No pred/No horm	-0.02769	0.0188	78	-1.469	0.8662	NS	
Int pred/No horm - No pred/CORT	-0.05082	0.0188	78	-2.697	0.1661	NS	
Int pred/No horm - No pred/MTP	0.01064	0.0188	78	0.564	0.9997	NS	
Int pred/No horm - No pred/No horm	0.01902	0.0188	78	1.009	0.9839	NS	
**No pred/CORT - No pred/MTP**	**0.06146**	**0.0188**	**78**	**3.261**	**0.0413**	**NS**	
**No pred/CORT - No pred/No horm**	**0.06984**	**0.0188**	**78**	**3.706**	**0.0112**	**NS**	
No pred/MTP - No pred/No horm	0.00838	0.0188	78	0.445	1	NS	

Supplementary Table S2. Pairwise contrasts for activity level among treatments during each day of observation.

Rows with an uncorrected P value < 0.05 are marked in bold. The rightside column indicates significance under the Bonferroni-corrected alpha (S: significant, NS: non-significant).

Day 0						
Contrast	Estimate	SE	df	t ratio	*P*	(Bonferroni-corrected alpha = 0.003)
Con pred/CORT - Con pred/MTP	-0.935	1.66	54	-0.562	0.993	NS
Con pred/CORT - Con Pred/No horm	-3.517	1.66	54	-2.113	0.2963	NS
Con pred/CORT - No pred/CORT	-2.07	1.66	54	-1.243	0.8136	NS
Con pred/CORT - No pred/MTP	-4.215	1.66	54	-2.532	0.1329	NS
Con pred/CORT - No pred/No horm	0.179	1.66	54	0.108	1	NS
Con pred/MTP - Con Pred/No horm	-2.582	1.66	54	-1.551	0.6336	NS
Con pred/MTP - No pred/CORT	-1.134	1.66	54	-0.681	0.9833	NS
Con pred/MTP - No pred/MTP	-3.28	1.66	54	-1.97	0.3724	NS
Con pred/MTP - No pred/No horm	1.115	1.66	54	0.67	0.9846	NS
Con Pred/No horm - No pred/CORT	1.448	1.66	54	0.87	0.9521	NS
Con Pred/No horm - No pred/MTP	-0.699	1.66	54	-0.42	0.9982	NS
Con Pred/No horm - No pred/No horm	3.696	1.66	54	2.22	0.2456	NS
No pred/CORT - No pred/MTP	-2.146	1.66	54	-1.289	0.7896	NS
No pred/CORT - No pred/No horm	2.248	1.66	54	1.351	0.7555	NS
No pred/MTP - No pred/No horm	4.394	1.66	54	2.64	0.1051	NS
Day 4						
Contrast	Estimate	SE	df	t ratio	*P*	(Bonferroni-corrected alpha = 0.003)
**Con pred/CORT - Con pred/MTP**	**-5.274**	**1.66**	**54**	**-3.168**	**0.0288**	**S**
Con pred/CORT - Con Pred/No horm	-0.128	1.66	54	-0.077	1	NS
**Con pred/CORT - No pred/CORT**	**-9.019**	**1.66**	**54**	**-5.418**	**<0.0001**	**S**
**Con pred/CORT - No pred/MTP**	**-9.643**	**1.66**	**54**	**-5.793**	**<0.0001**	**S**
**Con pred/CORT - No pred/No horm**	**-7.719**	**1.66**	**54**	**-4.637**	**0.0003**	**S**
**Con pred/MTP - Con Pred/No horm**	**5.146**	**1.66**	**54**	**3.092**	**0.0352**	**NS**
Con pred/MTP - No pred/CORT	-3.745	1.66	54	-2.25	0.2328	NS
Con pred/MTP - No pred/MTP	-4.37	1.66	54	-2.625	0.1087	NS
Con pred/MTP - No pred/No horm	-2.445	1.66	54	-1.469	0.6851	NS
**Con Pred/No horm - No pred/CORT**	**-8.892**	**1.66**	**54**	**-5.342**	**<0.0001**	**S**
**Con Pred/No horm - No pred/MTP**	**-9.516**	**1.66**	**54**	**-5.717**	**<0.0001**	**S**
**Con Pred/No horm - No pred/No horm**	**-7.591**	**1.66**	**54**	**-4.561**	**0.0004**	**S**
No pred/CORT - No pred/MTP	-0.625	1.66	54	-0.375	0.999	NS
No pred/CORT - No pred/No horm	1.3	1.66	54	0.781	0.9696	NS
No pred/MTP - No pred/No horm	1.925	1.66	54	1.156	0.8553	NS

Day 8							
Contrast	Estimate	SE	df	t ratio	*P*	(Bonferroni-corrected alpha = 0.003)	
Con pred/CORT - Con pred/MTP	0.989	1.66	54	0.594	0.991	NS	
Con pred/CORT - Con Pred/No horm	1.755	1.66	54	1.054	0.897	NS	
Con pred/CORT - No pred/CORT	-4.357	1.66	54	-2.618	0.1105	NS	
Con pred/CORT - No pred/MTP	-4.261	1.66	54	-2.56	0.1253	NS	
**Con pred/CORT - No pred/No horm**	**-5.589**	**1.66**	**54**	**-3.358**	**0.0172**	**NS**	
Con pred/MTP - Con Pred/No horm	0.766	1.66	54	0.46	0.9973	NS	
**Con pred/MTP - No pred/CORT**	**-5.346**	**1.66**	**54**	**-3.212**	**0.0257**	**NS**	
**Con pred/MTP - No pred/MTP**	**-5.25**	**1.66**	**54**	**-3.154**	**0.0299**	**NS**	
**Con pred/MTP - No pred/No horm**	**-6.579**	**1.66**	**54**	**-3.952**	**0.003**	**S**	
**Con Pred/No horm - No pred/CORT**	**-6.112**	**1.66**	**54**	**-3.672**	**0.007**	**NS**	
**Con Pred/No horm - No pred/MTP**	**-6.016**	**1.66**	**54**	**-3.614**	**0.0082**	**NS**	
**Con Pred/No horm - No pred/No horm**	**-7.345**	**1.66**	**54**	**-4.412**	**0.0007**	**S**	
No pred/CORT - No pred/MTP	0.096	1.66	54	0.058	1	NS	
No pred/CORT - No pred/No horm	-1.232	1.66	54	-0.74	0.9759	NS	
No pred/MTP - No pred/No horm	-1.328	1.66	54	-0.798	0.9667	NS	

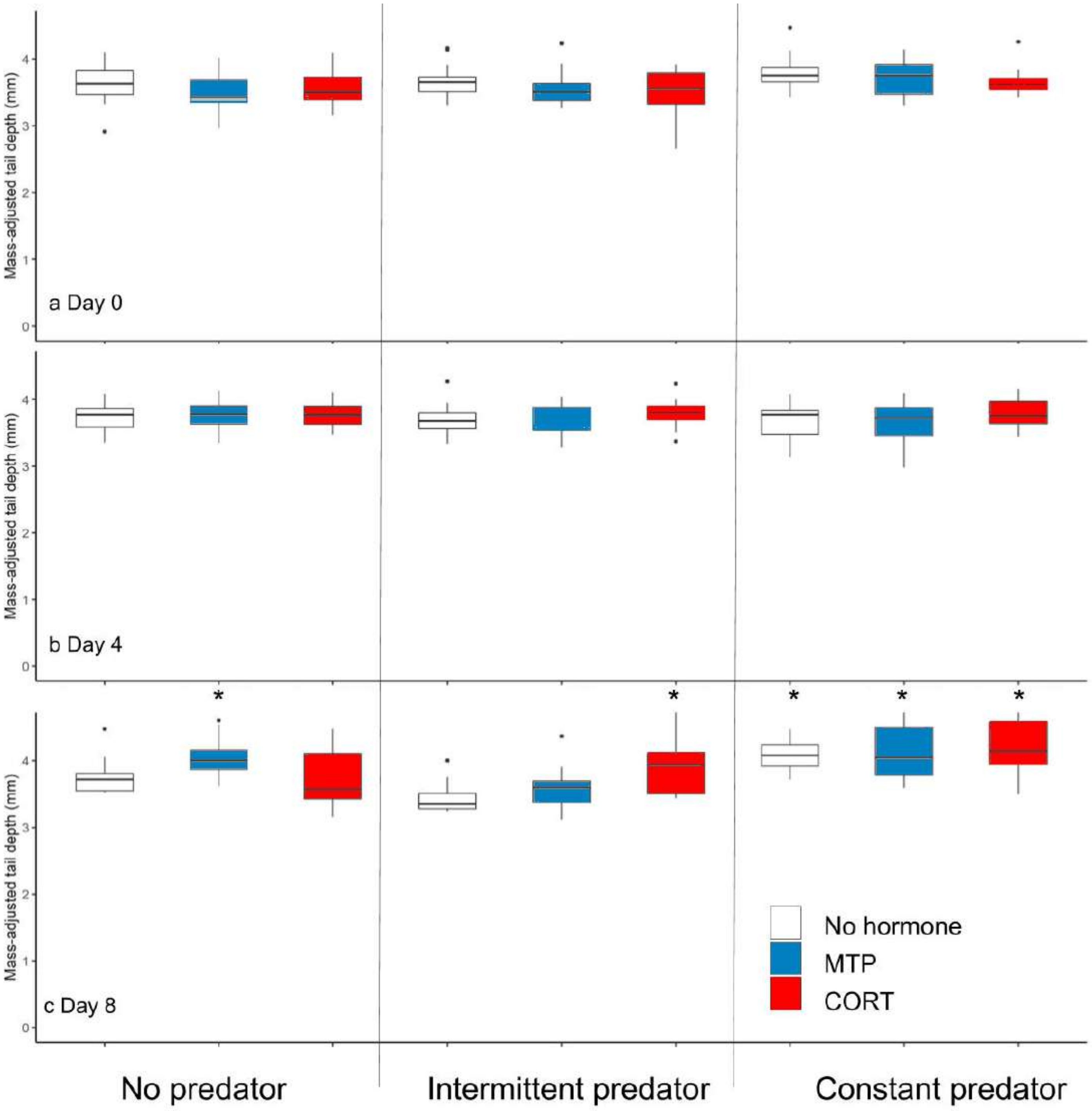

Supplementary Figure S1. Boxplots (median and interquartile range) of mass-adjusted tail depth of wood frog tadpoles in each treatment combination (*n* = 4 replicates) on Day 0 (a), Day 4 (b), and Day 8 (c). Asterisks indicate statistically significant differences compared to the No predator/No hormone treatment ($P < 0.05$).
